# A Novel, Fully Spliced, Accessory Gene in Equine Lentivirus with Distinct Rev-Responsive Element

**DOI:** 10.1128/jvi.00986-22

**Published:** 2022-09-07

**Authors:** Xiangmin Zhang, Jiwei Li, Mengmeng Zhang, Bowen Bai, Weiwei Ma, Yuezhi Lin, Xing Guo, Xue-Feng Wang, Xiaojun Wang

**Affiliations:** a State Key Laboratory of Veterinary Biotechnology, Harbin Veterinary Research Institute of Chinese Academy of Agricultural Sciences, Harbin, China; Icahn School of Medicine at Mount Sinai

**Keywords:** EIAV, HIV-1, Mat, RRE, Rev, lentiviruses, transcript, transcriptional regulation

## Abstract

All lentiviruses encode the accessory protein Rev, whose main biological function is to mediate the nuclear export of unspliced and incompletely spliced viral transcripts by binding to a viral cis-acting element (termed the Rev-responsive element, RRE) within the env-encoding region. Equine infectious anemia virus (EIAV) is a member of the lentivirus genus in the Retroviridae family and is considered an important model for the study of lentivirus pathogenesis. Here, we identified a novel transcript from the EIAV genome that encoded a viral protein, named Mat, with an unknown function. The transcript *mat* was fully spliced and comprised parts of the coding regions of MA and TM. Interestingly, the expression of Mat depended on Rev and the chromosome region maintenance 1 (CRM1) pathway. Rev could specifically bind to Mat mRNA to promote its nuclear export. We further identified that the first exon of Mat mRNA, which was located within the Gag-encoding region, acted as an unreported RRE. Altogether, we identified a novel fully spliced transcript *mat* with an unusual RRE, which interacted with Rev for nuclear export through the CRM1 pathway. These findings updated the EIAV genome structure, highlighted the diversification of posttranscriptional regulation patterns in EIAV, and may help to expand the understanding of gene transcription and expression of lentivirus.

**IMPORTANCE** In lentiviruses, the nuclear export of viral transcripts is an important step in controlling viral gene expression. Generally, the unspliced and incompletely spliced transcripts are exported via the CRM1-dependent export pathway in a process mediated by the viral Rev protein by binding to the Rev-responsive element (RRE) located within the Env-coding region. However, the completely spliced transcripts are exported via an endogenous cellular pathway, which was Rev independent. Here, we identified a novel fully spliced transcript from EIAV and demonstrated that it encoded a viral protein, termed Mat. Interestingly, we determined that the expression of Mat depended on Rev and identified that the first exon of Mat mRNA could specifically bind to Rev and be exported to the cytoplasm, which suggested that the first exon of Mat mRNA was a second RRE of EIAV. These findings provided important insights into the Rev-dependent nuclear export of completely spliced transcripts in lentiviruses.

## INTRODUCTION

Equine infectious anemia virus (EIAV) is a member of the lentivirus genus of the Retroviridae family and primarily infects *equids*. Unlike other lentiviral infections characterized by chronic degenerative progression, EIAV infection in horses usually leads to an acute disease course (1). Moreover, after approximately 1 year of infection, most infected animals can control viral replication and progress to inapparent carriers (2). An inapparent carrier can still recrudesce disease under stress or immune suppression (1, 3). These phenomena indicate that infected horses have gained immunologic control over the EIAV. Thus, the EIAV system provides a good model for the study of lentivirus replication and lentiviral vaccine development (4).

Lentiviruses, including the human immunodeficiency virus type 1 (HIV-1), simian immunodeficiency virus (SIV), and EIAV, like all retroviruses, are RNA viruses that replicate via a DNA intermediate (called proviral DNA), which is integrated into the host genomic DNA. In addition to the three structural proteins (Gag, Pol, and Env) common to retroviruses, the lentiviral genome also encodes several accessory proteins. Current knowledge indicates that EIAV encodes at least four accessory proteins (Tat, S2, Rev, and Ttm) (5), while primate lentiviruses (HIV-1 and SIV) encode six accessory proteins (Tat, Rev, Nef, Vpu, Vif, and Vpr/Vpx) (6, 7). Thus, EIAV is considered to be the lentivirus with the simplest genome organization (1, 5). These accessory proteins are not only directly involved in viral transcriptional and posttranscriptional regulation (Tat and Rev) (8), but also regulate various stages of viral replication by interacting with host proteins (e.g., Nef, Vpu, Vif, Vpr, and S2) (9–15). In particular, the accessory proteins antagonizing host restriction factors to promote viral replication in HIV-1 have been well studied (9, 16, 17). However, how EIAV utilizes fewer viral proteins to antagonize those diverse host restriction factors is an intriguing question. Perhaps there are additional viral proteins that have not been identified in EIAV, but this speculation requires further investigation. At both ends of the lentiviral genome are identical long terminal repeats (LTR) (18). As a viral promoter, 5' LTR regulates viral genome transcription by binding with host RNA polymerase II, other host proteins (such as cyclinT) as well as virus-encoded proteins, including Tat (8, 19).

The lentiviral proviral DNA acts as a transcription template to synthesize viral mRNA in the nucleus. Transcription is initiated at the U3-R border of the 5′ LTR and terminates at the R-U5 border of the 3′ LTR (20). A full-length genome mRNA is first transcribed, which is a primary unspliced transcript, and then most of this transcript undergoes alternative splicing to generate multiple spliced transcripts, allowing the expression of several viral proteins. In HIV-1, more than 40 transcripts have been found, which are responsible for the expression of the viral proteins (8). In EIAV, five transcripts have been identified in earlier studies (5, 21–23), including an unspliced full-length mRNA transcript encoding Gag and Pol protein as well as genomic RNA for encapsidation into progeny virions, an incompletely spliced transcript encoding Env and S2 protein, three fully spliced subgenomic transcripts encoding the accessory proteins (Tat, Rev), and a predicted Ttm protein whose function is unknown. However, it is not clear whether EIAV could express more transcripts like HIV-1.

In lentiviruses, the synthesis and production of all viral mRNA take place in the nucleus and the mRNA is then transported to the cytoplasm by different nuclear export pathways (24). For example, in HIV-1, the unspliced full-length and incompletely spliced transcripts encoding viral structural proteins (Gag, Pol, and Env) and some accessory proteins (vif, vpr, and vpu) are exported to the cytoplasm utilizing the chromosome region maintenance 1 (CRM1)-dependent export pathway mediated by the viral Rev protein. However, all the completely spliced transcripts encoding the viral accessory proteins Tat, Rev, and Nef, are exported to the cytoplasm by an endogenous cellular pathway used by cellular mRNAs independent of Rev (8, 25). Other lentiviruses, such as EIAV and FIV, also utilize the Rev-mediated CRM1 pathway to export incompletely spliced mRNA transcripts (26). The Rev protein is an accessory protein of all lentiviruses and interacts with a specific cis-acting element termed the Rev responsive element (RRE) at the viral pre-mRNA (27, 28). The lentiviral RRE is a highly structured viral RNA sequence that is generally located in the Env-coding region (27). Although the lentiviral RREs lack sequence homology, they may share specific stem-loop structures. Some studies have identified that the RRE in HIV-1 is about 350 nt and that in EIAV is about 555 nt (27, 29). The molecular details of the Rev-mediated incompletely spliced mRNA export have been well characterized (30). A single Rev molecule first binds to RRE, and subsequently, multiple Rev molecules undergo a series of Rev-Rev oligomerizations to form a multimer, which subsequently recruits the nuclear export factor CRM1 to promote the export of the incompletely spliced mRNA into the cytoplasm. Thus, Rev binding and subsequent multimerization are necessary for the Rev-RRE complex function. Previous studies have identified the RNA binding domain and multimerization of EIAV Rev (31–34). Considering that Rev-mediated RNA export is essential for lentiviral replication, the Rev-RRE complex is a potential therapeutic target in lentiviruses.

In the present study, we discovered a novel viral protein in EIAV and named it Mat. The protein was translated from an unreported fully spliced transcript from the EIAV genome. Interestingly, the expression of Mat depended on the Rev-mediated RNA nuclear export pathway. We provided evidence that Rev bound to Mat mRNA and was required for the nuclear export of Mat mRNA.

## RESULTS

### Discovery and identification of a novel protein-encoding gene from EIAV.

**FIG 1:**
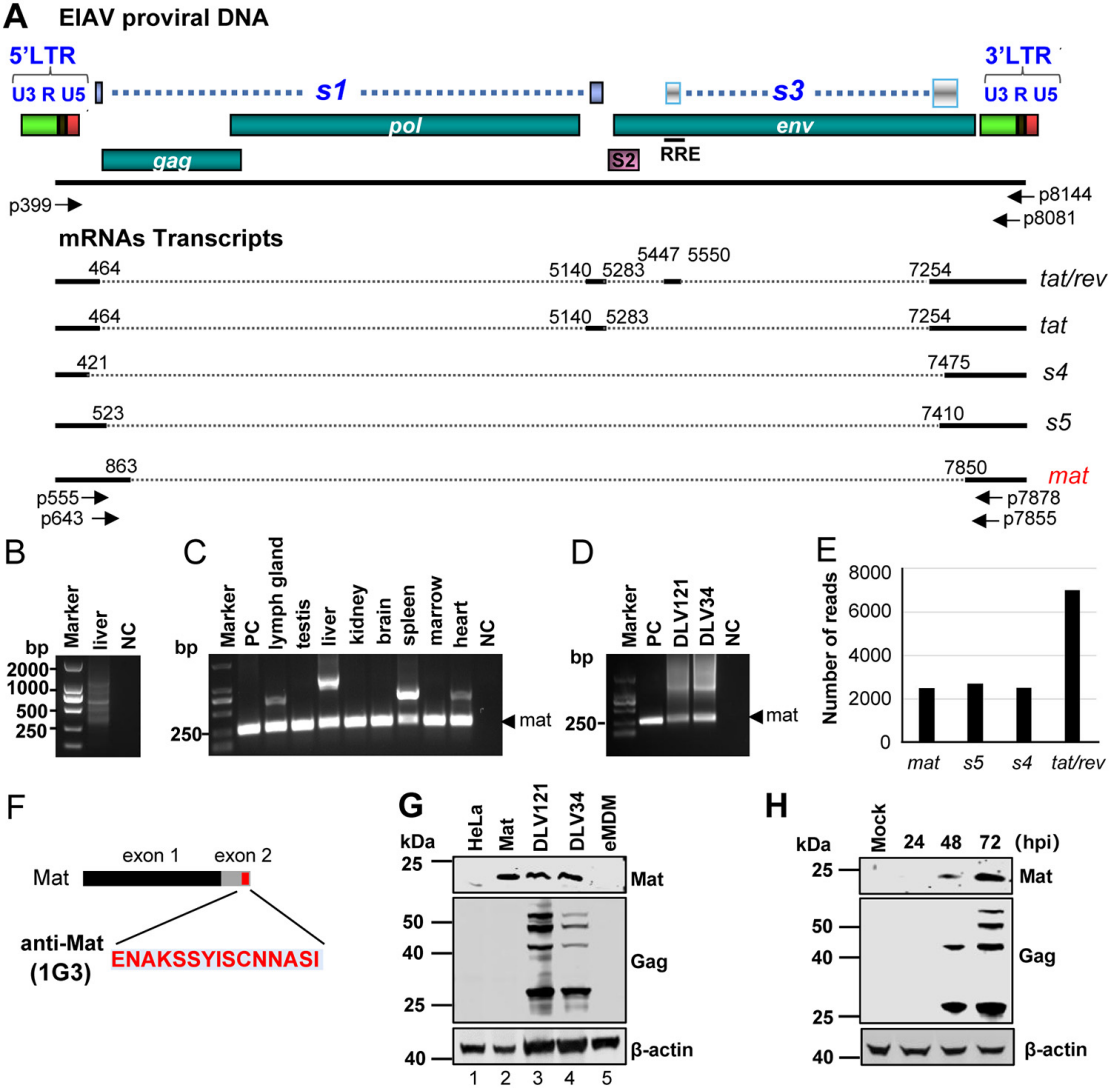
Discovery and identification of a novel protein-encoding gene from EIAV. (A) A schematic representation of the EIAV genome is displayed at the top of the figure. Known ORFs are shown as boxes with different colors. The position of RRE is depicted with a heavy line. The splicing patterns of EIAV transcripts identified in this study are shown below the genomic structure. The numbers represent splice sites of mRNA. Horizontal arrows show the locations of oligonucleotide primers for the amplification of cDNAs. (B) RT-PCR analysis of total RNAs extracted from horse liver infected with EIAV_LN40_. cDNAs were amplified using the nested PCR primer pairs p399/p8144, and p399/p8081, as indicated. PCR products were resolved on an agarose gel. (C) Identification of the mat gene from multiple tissues (lymph gland, testis, liver, kidney, brain, spleen, marrow, and heart) of horses infected with EIAV_LN40_ by RT-PCR using the nested PCR primer pairs p555/p7878, and p643/p7855, as indicated. PC, mat sequence inserted into pMD18T-vector as a positive control. NC, negative control. (D) Identification of the mat gene from eMDM infected with EIAV_DLV34_ and EIAV_DLV121_ by RT-PCR. (E) Identification of EIAV transcripts from eMDM infected with EIAV by RNA-seq. The vertical axis shows the number of reads with mat (AATGATTGATG↑ATTGGGAAAA)-, s4 (GAACTTACAGACG↑GCAAAAGA)-, s5 (GAAGGTGACGG↑GACAAGTCCA)-, and tat/rev (TCTGTTATAAG↑CCATAAAGCA)-specific splicing patterns from an RNA-seq sequence library derived from eMDM infected with EIAV_DLV121._ (F) Schematic representation for an epitope of the anti-Mat monoclonal antibody 1G3. (G) Identification of Mat from eMDM infected with EIAV_DLV34_ and EIAV_DLV121_. The Mat-specific antibody 1G3 (described in Materials and Methods) was used for the immunodetection of Mat protein. An anti-EIAV p26 antibody was used to monitor the effect of infection. HeLa cells transfected with empty plasmids were used as a negative control (lane 1). HeLa cells transfected with a codon-optimized Mat expression plasmid were used as a positive control (lane 2). eMDM cells isolated from healthy horses were used as a specific control (lane 5). (H) Time course analyses of Mat expression. eMDM cells were infected with EIAV_DLV121_ at an MOI of 5. At each indicated time point, total cell lysates were analyzed by Western blotting with an anti-p26 antibody, and an anti-Mat antibody 1G3. hpi, hours postinoculation.

EIAV has long been considered the lentivirus with the simplest genome organization (1, 5), mainly producing five transcripts encoding seven viral proteins, which were discovered in studies more than 30 years ago (21–23). To test whether EIAV further encodes other unidentified transcripts, we designed an upstream primer (p399), and two downstream primers (p8144 and p8081) based on the genome structure and transcriptional characteristics of EIAV, as shown in Fig. 1A. RNA extracted from EIAV-infected horse liver was amplified using RT-PCR as per the procedures described in Materials and Methods. The products of PCR were loaded onto a 1% agarose gel and there were multiple blurred bands below 1 kb (Fig. 1B). The PCR products were cloned and sequenced, and the sequences were then aligned to the EIAV genome (GenBank accession number GU385361.1). A total of five EIAV-specific mRNAs were discovered by sequence alignment, including the previously reported tat/rev and tat mRNAs and three novel mRNAs (s4, s5, and mat) (Fig. 1A). In this study, we focused on one of these novel transcripts (GenBank accession number OM417133), which was generated by a single-splicing event with a 5′ splice donor site (SD) at nt863 (^862^TG^863^|^864^GG^865^) and a 3′ splice acceptor site (SA) at nt7850 (^7848^AT^7849^|^7850^AT^7851^). The transcript included a 447 bp open reading frame (ORF), and contained two exons, with the first exon (393 bp) partially overlapping with the MA subunit coding region of the Gag protein (in the same reading frame) and the second exon (54 bp) partially overlapping the 3’terminal region of the TM coding region of the Env protein (in a different reading frame). Therefore, we named the novel encoding gene *mat* and its encoding protein Mat. According to the splicing pattern of the mat transcripts, we designed specific nested PCR primers (forward primers, p555 and p643; reverse primers, p7878 and p7855) to amplify part of this transcript sequence (about 250 bp) from multiple tissues (lymph gland, testis, liver, kidney, brain, spleen, marrow, and heart) of horses infected with EIAV_LN40_ (Fig. 1C). The obtained RT-PCR products were sequenced and the junction between the SD^863^ and SA^7850^ sites, namely, mat-specific splicing sites was found. We also amplified the mat-specific bands from the equine monocyte-derived macrophages (eMDM) infected with EIAV_DLV121_ and EIAV_DLV34_ *in vitro* (Fig. 1D). To further confirm the production of mat transcript from EIAV, we aligned mat-, s4-, s5-, and tat/rev-specific sequences with transcriptomic data from EIAV-infected eMDMs obtained by RNA-seq (35) and found mat-specific reads and other EIAV-specific reads (Fig. 1E) that were not found in uninfected eMDMs. All these data suggested that there were *mat* transcripts in tissues or cells infected with EIAV. Furthermore, to detect the expression of Mat in EIAV-infected cells, we generated a mouse monoclonal antibody (1G3) against the peptide (ENAKSSYISCNNASI), which uniquely corresponded to the translated product of the second exon of mat but no other known viral proteins (Fig. 1F). Using this antibody, we detected an about 20-kDa specific band in eMDM cells infected with EIAV_DLV121_ and EIAV_DLV34_, but not in uninfected cells (Fig. 1G). In addition, we also observed that Mat expression increased gradually with time after EIAV infected eMDM cells (Fig. 1H). These results suggested that the Mat protein was expressed in EIAV-infected cells.

### The expression of Mat depended on the nuclear export activity of Rev.

**FIG 2:**
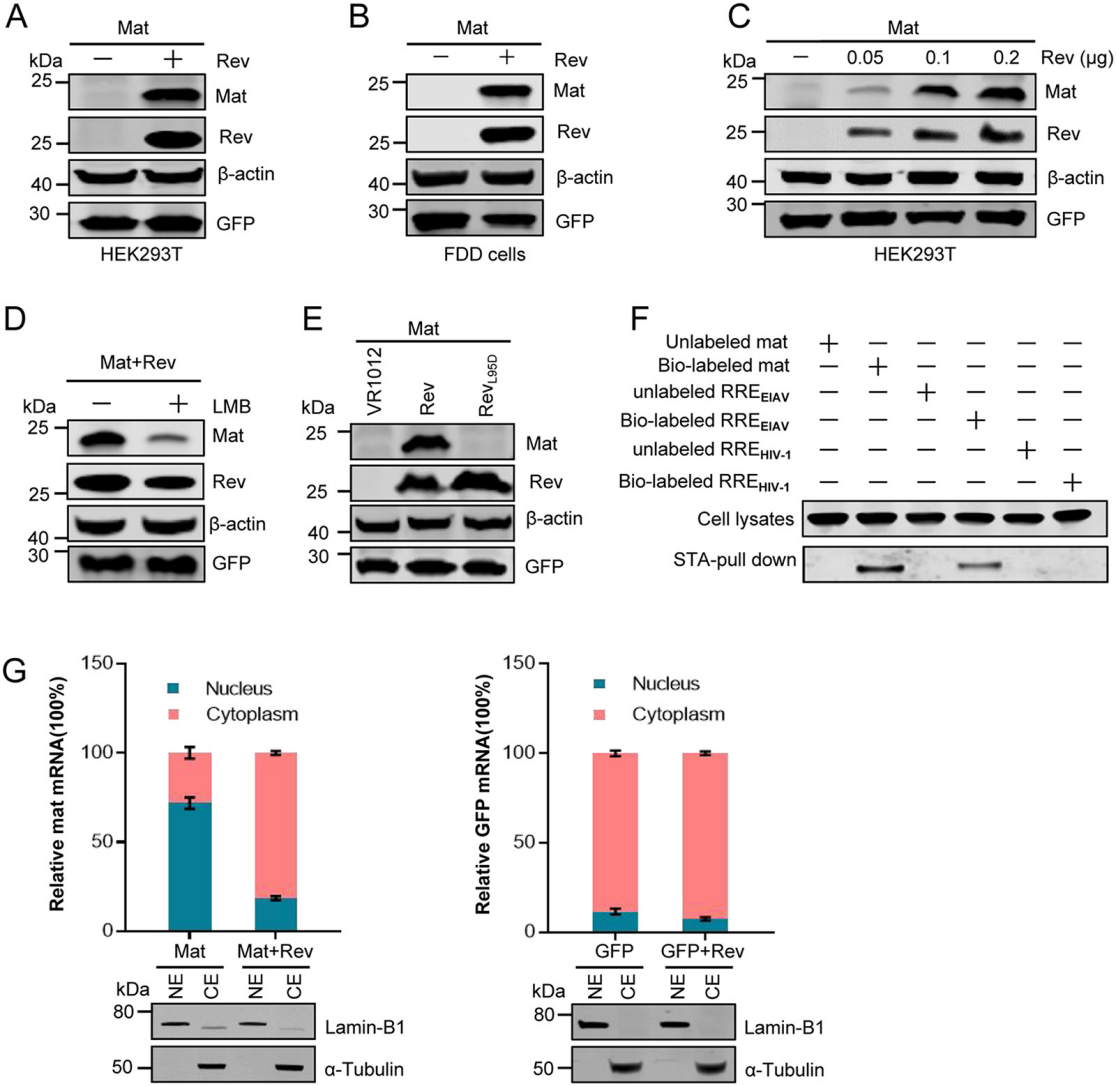
The expression of Mat depended on Rev. A Mat constructs were transiently cotransfected into HEK293T cells (A) or FDD cells (B) with Rev expression plasmids or empty plasmids. Mat expression was analyzed with WB using a mouse anti-HA antibody 48 h posttransfection (hpt); β-actin expression was detected using a mouse anti-actin antibody to monitor unity of blotting and equal loading of the gel; GFP expression was quantified using a mouse anti-GFP antibody to monitor transfection efficiencies. (C) A Mat construct was transfected into HEK293T cells, along with increasing amounts of a Rev expression plasmid. (D) LMB inhibited the Rev-independent expression of Mat. HEK293T cells were cotransfected with Mat constructs and Rev expression plasmids. At 12 hpt, cells were treated with leptomycin B (0 nM [−] or 50 nM [+], as indicated). At 24 h post drug treatment, cells were harvested and subjected to WB. (E) No Mat protein expression was detected when cotransfected with Mat constructs and Rev_L95D_ expression plasmids. Rev_L95D_ was a mutant of Rev without the ability to mediate mRNA export to the cytoplasm. VR1012 was used as empty vector control. (F) Interaction between mat mRNA and Rev protein was determined using an RNA-pulldown assay. Cell lysates from HEK293T transfected with Rev expression plasmids were incubated with biotin-labeled or unlabeled RNA transcribed *in vitro*. The biotin-labeled mat mRNA, EIAV RRE or HIV-1 RRE were pulled down with streptavidin (STA)-conjugated magnetic beads, followed by WB of copurified proteins using anti-Flag antibody. (G) The distribution of cytoplasmic and nuclear mat or GFP mRNAs in HEK293T cells at 36 hpt was analyzed using qPCR with primers specific to mat or GFP mRNAs. Ratios of cytoplasmic to nuclear mRNAs from the same sample were then calculated and presented. Error bars represent standard deviations (SD) of three independent experiments. LaminB1 and α-tubulin were detected using WB to assess the efficacy of cell fractionation. All the experiments were performed three times, and a representative result is shown.

To express the novel protein Mat in a eukaryotic expression system, its coding sequences were inserted into a pcDNA3.1 vector. We were unable to detect the expression of Mat when transfected HEK239T cells with the Mat expression plasmid alone. However, when HEK293T were cotransfected Mat with the Rev expression plasmid, Mat proteins could be observed using Western blotting (WB) (Fig. 2A). This Mat expression pattern was also observed in fetal donkey dermal (FDD) cells (Fig. 2B), which are permissive cells for the replication of EIAV (36, 37). GFP was used here as a specific control to monitor transfection efficiencies. This result suggested that Rev was necessary for the expression of Mat. Furthermore, when HEK293T cells were transfected with different doses of Rev, the expression of Mat increased in a dose-dependent manner (Fig. 2C).

It is well known that Rev is a nuclear-cytoplasmic shuttling protein that can facilitate the export of viral mRNAs from the nucleus via the CRM1-mediated pathway (8, 28). To investigate whether the expression of Mat was related to the nuclear export activity of Rev, HEK293T cells were cotransfected with Mat and Rev plasmids and then were treated with leptomycin B (LMB), which was an inhibitor of CRM1. As shown in Fig. 2D, the levels of Mat expression significantly decreased in the presence of LMB. In addition, a Rev mutant (Rev_L95D_), that disrupted nuclear export activity (33, 34), was unable to mediate the expression of Mat to the same extent as the wild-type Rev (Fig. 2E). These results indicated that Rev-mediated Mat expression depended on the nuclear export activity of Rev through the CRM1-pathway.

To assess whether mat mRNAs interact with Rev protein, an RNA-pulldown assay was performed using biotin-labeled mat RNA and streptavidin (STA) beads. WB analysis with anti-Flag antibody was used to detect whether any Rev protein was present in the cell lysates in the mat mRNA pulldown. The results suggested that EIAV Rev could specifically bind to mat and EIAV RRE mRNAs (reported RNA export element in EIAV, used as a positive-binding control) but not to HIV-1 RRE mRNAs (RNA export element in HIV-1, used as a specific-binding control) (Fig. 2F). Furthermore, we isolated nuclear and cytoplasmic RNAs from HEK293T cells after cotransfection with Mat and Rev or empty plasmids, and the RNAs were subsequently analyzed using quantitative PCR (qPCR). Our results showed that most of the mat mRNAs were retained in the nucleus in the absence of Rev, whereas the proportion of mat mRNAs in the cytoplasm increased markedly when cotransfected with Rev (Fig. 2G, left). In contrast, the presence of Rev made no difference to the nucleocytoplasmic ratio of GFP mRNAs (Fig. 2G, right). These results implied that Rev could specifically promote the export of mat mRNAs from the nucleus to the cytoplasm. To evaluate the efficiency of separation, LaminB1 and α-Tubulin protein were used as controls for nuclear and cytoplasmic proteins, respectively (Fig. 2G, bottom). All these results together demonstrated that the expression of Mat depended on Rev, and Rev could promote the transport of mat mRNAs by binding to them.

### The first exon of mat was necessary for the Rev-mediated expression of Mat.

**FIG 3:**
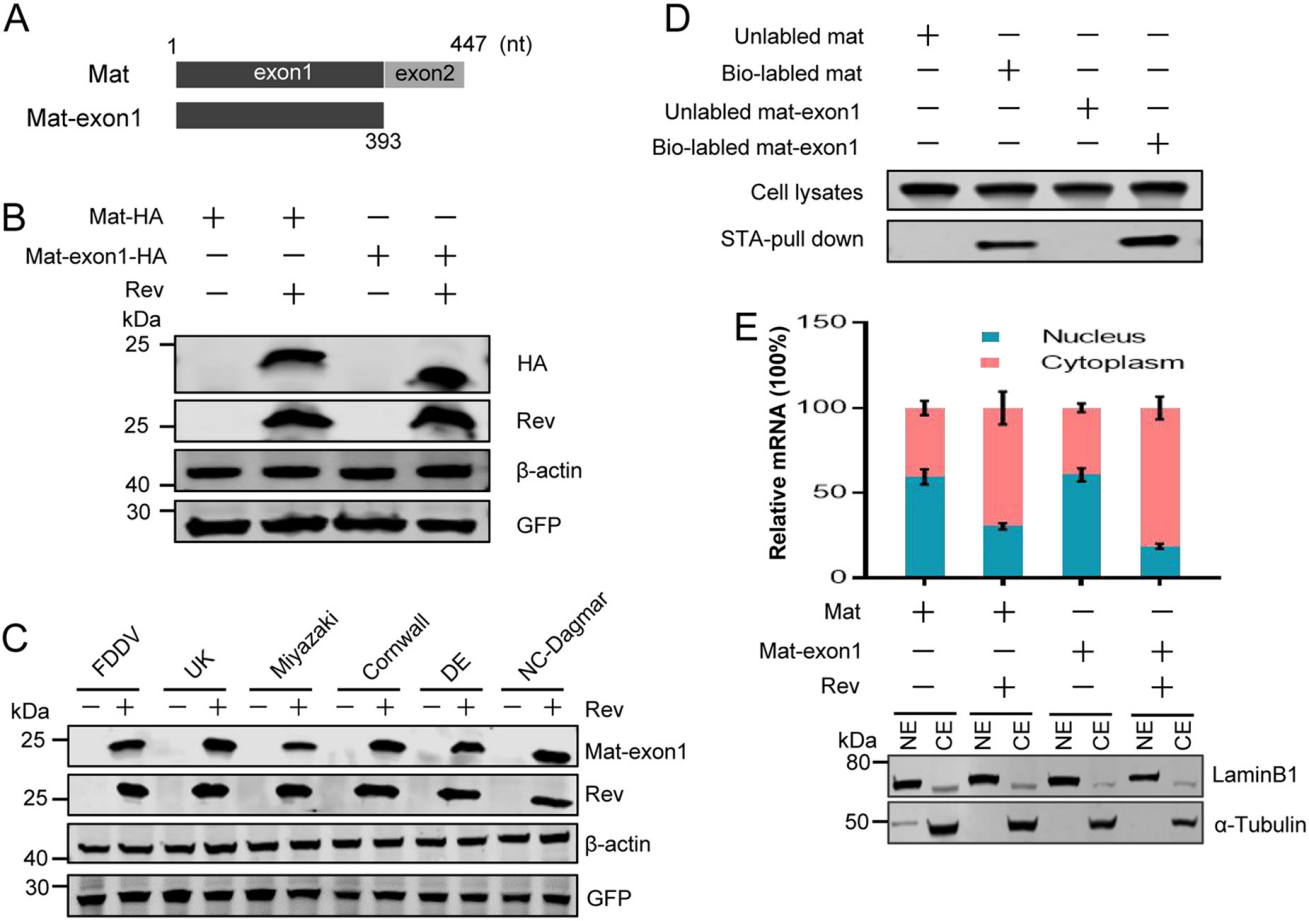
Mapping of the determinants of Rev-mediated expression of Mat. (A) Schematic diagram of the mat ORF coding sequence with 447 nucleotides, which contained two exons (exon-1, 1 to 393 nt; exon-2, 394 to 447 nt). The exon-1 sequence was inserted into pcDNA-HA to express the Mat-exon1 protein. (B) HEK293T cells were cotransfected Mat or mat-exon1 expression constructs with Rev or empty plasmids. Mat expression was analyzed with WB using a mouse anti-HA antibody; GFP, derived from pEGFP-N1, was quantified using a mouse anti-GFP antibody to monitor transfection efficiencies. (C) HEK293T cells were cotransfected each of six different Mat-exon1 plasmids, which were derived from six EIAV strains as indicated, with Rev or empty plasmids. Mat-exon1 proteins were detected 48 hpt with WB. (D) An RNA-pulldown assay was performed to verify the interaction of mat-exon1 mRNA with Rev protein. The Rev pulled down by mat mRNA acted as a positive control. Biotin-unlabeled mRNAs were used to indicate the specificity of this assay. (E) The distribution of cytoplasmic and nuclear mat-exon1 mRNAs in HEK293T cells at 36 hpt was analyzed using qPCR. The subcellar distribution of mat mRNAs was analyzed as a control. Error bars represent the SD of three independent experiments.

The ORF of mat contained two exons, with the second one having only 54 nucleotides. To map the determinants of Mat expression more precisely, we constructed a Mat-exon1 expression plasmid (Fig. 3A). As expected, we found that the expression of Mat-exon1 also depended on Rev, which was consistent with the expression characteristics of Mat (Fig. 3B). To further confirm this phenomenon in multiple EIAV strains, we constructed 6 mat-exon1 expression plasmids with DNA sequences, each corresponding to the N terminus of the *gag* gene coding sequence from one of the different strains, as described in Materials and Methods. We then cotransfected these mat-exon1 expression plasmids either with Rev or with an empty plasmid. WB analysis demonstrated that, in all cases, Mat-exon1 was expressed only in the presence of Rev (Fig. 3C). It further confirmed that Rev was necessary for Mat-exon1 expression in the different EIAV strains. We then used an RNA pulldown assay to assess the interaction between mat-exon1 mRNA and Rev. The results demonstrated that mat-exon1 mRNA could also specifically bind to Rev (Fig. 3D). In addition, we separated the cytoplasm and nucleus of the transfected cells and quantified the RNAs in the different fractions separately. The qPCR results showed that accumulation of mat-exon1 mRNAs in the cytoplasm increased in the presence of Rev (Fig. 3E). These results identified the first exon of mat as being the determinant of Rev-mediated Mat expression, with the first exon demonstrating the same characteristics as the full-length mat mRNAs in terms of protein expression, Rev binding, and nucleocytoplasmic transport of mRNAs.

### Different key domains of Rev underlie the mechanism by which the expression of Gag and Mat was regulated.

**FIG 4:**
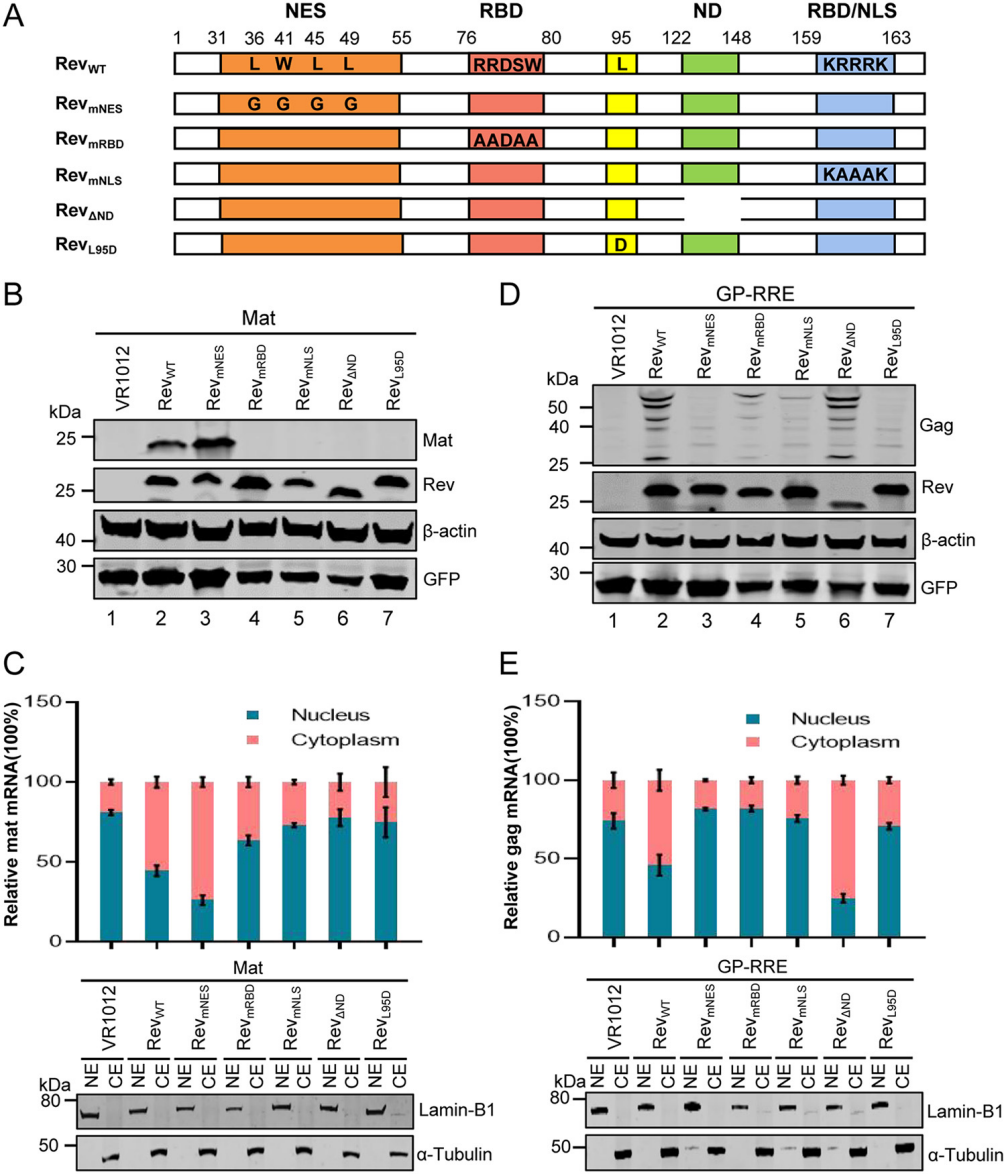
The key domains of Rev mediating the expression of Gag and Mat were different. (A) Schematic diagram of wild-type EIAV Rev, which contained four functional domains: NES (nuclear export domain; aa 31 to 55); RBD (RNA-binding domain; aa76-88 or aa159-163); ND (Nonessential domain; aa122-148); NLS (nuclear localization domain; aa159-163). The structures of Rev five mutants were Rev_mNES_, the mutation of Rev from L36, W41, L45, and L49 to G; Rev_mRBD_, the mutation of Rev from RRDSW to AADAA; Rev_mNLS_, the mutation of Rev from KRRRK to KAAAK; Rev_ΔND_, the 27 amino acid (aa122-148) deletion of ND domain and Rev_L95D_, described in the Materials and Methods. WB analysis of Mat (B) or Gag (D) expression levels when a Mat expression plasmid or a Rev-dependent EIAV Gag/Pol expression construct (pGP-RRE) was transfected into HEK293T cells with either an empty vector VR1012 (as a negative control) or a series of Rev expression plasmids, as indicated. The distribution of cytoplasmic and nuclear mat (C) or gag (E) mRNAs in HEK293T cells at 36 pht was analyzed using qPCR when Mat or pGP-RRE expression plasmids were transfected into HEK293T cells with a series of Rev expression plasmids. Error bars represent the SD of three independent experiments. All the experiments were performed three times, and a representative result is shown.

Previous studies have described the four functional domains within Rev: a nuclear export signal (NES), an RNA binding domain (RBD), a nuclear localization signal (NLS), and a functionally unknown nonessential domain (ND) (38). To map the key domain responsible for the Rev mediation of Mat expression, we constructed five Rev mutants, including the four mutants with defects in the respective functional domains (39), and a multimerization-defective mutant Rev_L95D_ that lost the capacity to promote the export of the gag-pol mRNAs from the nucleus to the cytoplasm (34) (Fig. 4A). A Mat expression vector was cotransfected into HEK293T cells with each Rev mutant separately. After 48 h, the level of Mat protein was assessed using WB and the distribution of mat mRNAs in the cytoplasm and nucleus was analyzed using qPCR as the ratio of nuclear to cytoplasmic mat mRNA. The WB analysis results showed that only the Rev_WT_ and Rev_mNES_ were able to mediate the expression of Mat, while the other four mutants almost completely lost this function (Fig. 4B). The qPCR analysis also showed that mat mRNAs were mainly distributed in the cytoplasm in the presence of Rev_WT_ or Rev_mNES_, while the other Rev mutants showed results similar to the absence of Rev, where mat mRNA was mainly distributed in the nucleus (Fig. 4C). These results indicated that the RBD, NLS and ND domains of Rev and their multimerization played an important role in mediating the nuclear export of mat mRNAs.

In addition, a Rev-dependent EIAV Gag-Pol expression vector, pGP-RRE (described in Materials and Methods), was transfected into HEK293T cells in the presence of Rev_WT_ or different Rev mutants. The levels of Gag protein were assessed using WB and the distribution of gag mRNA in the cytoplasm and nucleus was analyzed using qPCR. No Gag protein was detected in the absence of Rev or the presence of Rev_L95D_ (Fig. 4D, lanes 1 and 7). Compared to those in the presence of Rev_WT_, little Gag protein was detected in the presence of Rev_mNES_, Rev_mRBD_, or Rev_mNLS_ (Fig. 4D, lanes 3, 4, and 5), but none of the Gag proteins expression levels showed remarkable changes in the presence of Rev_ΔND_ compared with the presence of Rev_WT_ (Fig. 4D, lane 6). The qPCR analysis also showed that gag mRNAs were mainly distributed in the cytoplasm in the presence of Rev_WT_ or Rev_ΔND_, while the presence of other Rev mutants gave results similar to those obtained in the absence of Rev, with the gag mRNAs being mainly distributed in the nucleus (Fig. 4E). Consistent with the results of previous studies (38), these results also indicate that in addition to ND, other functional domains of Rev play an important role in mediating the nuclear export of gag-pol mRNAs.

Taken together, these results showed that the expression levels of Mat or Gag protein were related to the accumulation of the mRNAs in the cytoplasm. Interestingly, we found that the export of mat and gag mRNAs from the nucleus relied on different functional domains of Rev, indicating that Rev mediated the expression of Gag and Mat through different mechanisms.

### The first exon of Mat could not replace the known RRE to mediate EIAV Gag/Pol expression.

**FIG 5:**
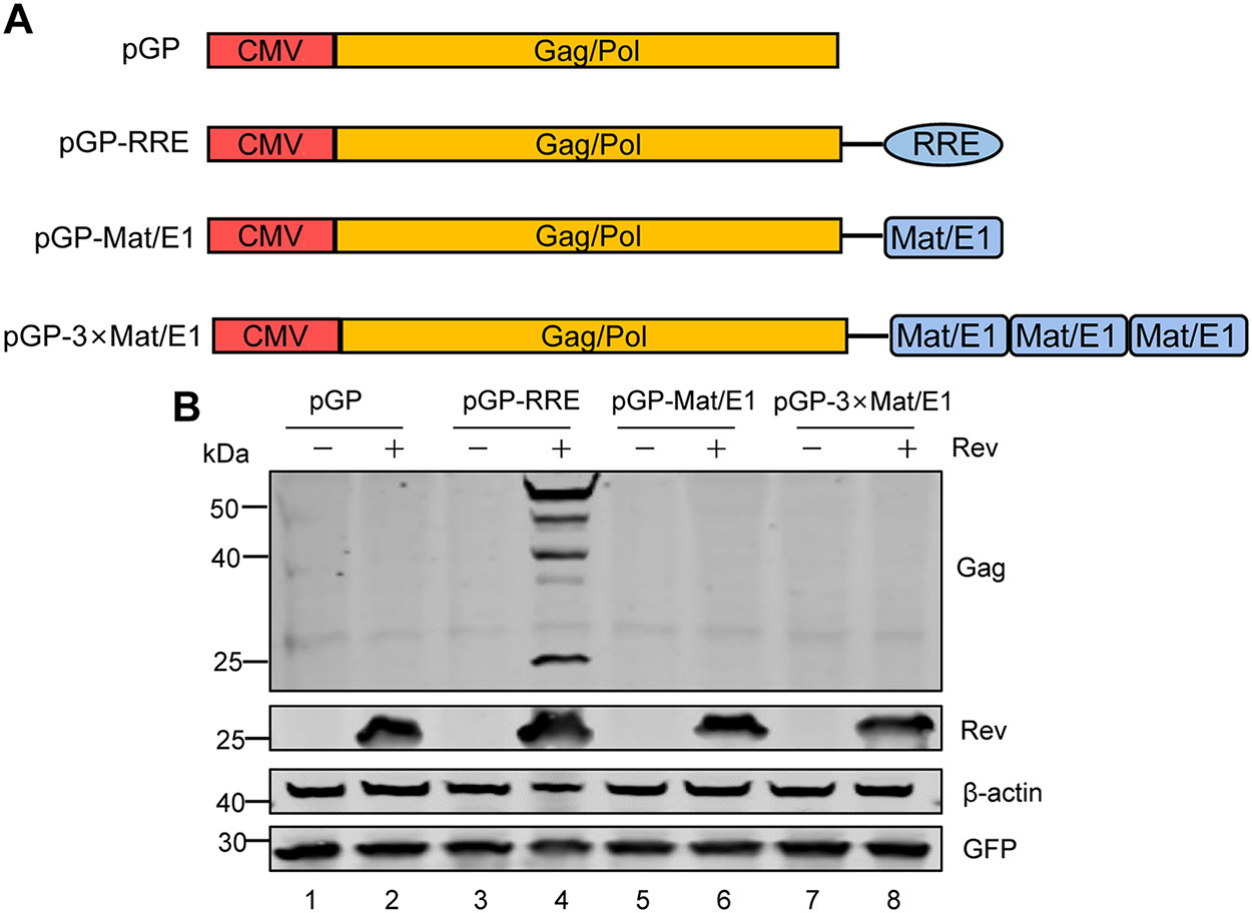
Functional identification of Mat's first exon used as an RNA export element. (A) Schematic diagram of a series of plasmids used for the identification of the function of the RNA export element. pGP, contained only EIAV gag/pol-coding sequences; pGP-RRE, with EIAV RRE inserted downstream of the gag/pol coding sequence; pGP-Mat/E1, with one copy of mat-exon1(Mat/E1) inserted downstream of the gag/pol-coding sequence; pGP-3×Mat/E1 with three copies of Mat/E1 inserted downstream of the gag/pol-coding sequence. (B) WB analysis of indicated plasmids cotransfected with Rev or empty vector. All the experiments were performed three times, and a representative result is shown.

As described above, the first exon of mat could be used as an RNA export element to mediate the expression of Mat by binding to Rev protein. Therefore, to test whether the mat-exon1 (Mat/E1) could replace the known RRE to mediate the expression of Gag/Pol, a series of EIAV Gag/Pol constructs was prepared: the pGP contained only the EIAV gag/pol-coding sequences; pGP-RRE, pGP-Mat/E1 and pGP-3×Mat/E1 were based on pGP with an EIAV RRE (22), one copy of Mat/E1, or three copies of Mat/E1 inserted, respectively, downstream of the gag/pol coding sequence (Fig. 5A). HEK293T cells were transfected with these constructs in the presence or absence of Rev, and the levels of Gag protein in the cells were analyzed using WB. Gag expression could only be detected in cells transfected with pGP-RRE in the presence of Rev (Fig. 5B, lane 4), while we could not detect Gag expression in cells transfected with other constructs with or without Rev. Therefore, these results indicated that the Mat first exon sequence within the Gag/pol-coding sequence was unable to mediate the expression of Gag/Pol under the effect of Rev (Fig. 5B, lane 2), and the Mat/E1 could not replace RRE to mediate the expression of Gag/Pol (Fig. 5B, lanes 6 and 8). Taken together, these results showed that single or multimer copies of mat-exon1 could not replace the RRE function in the EIAV Gag/Pol expression vector.

## DISCUSSION

**FIG 6:**
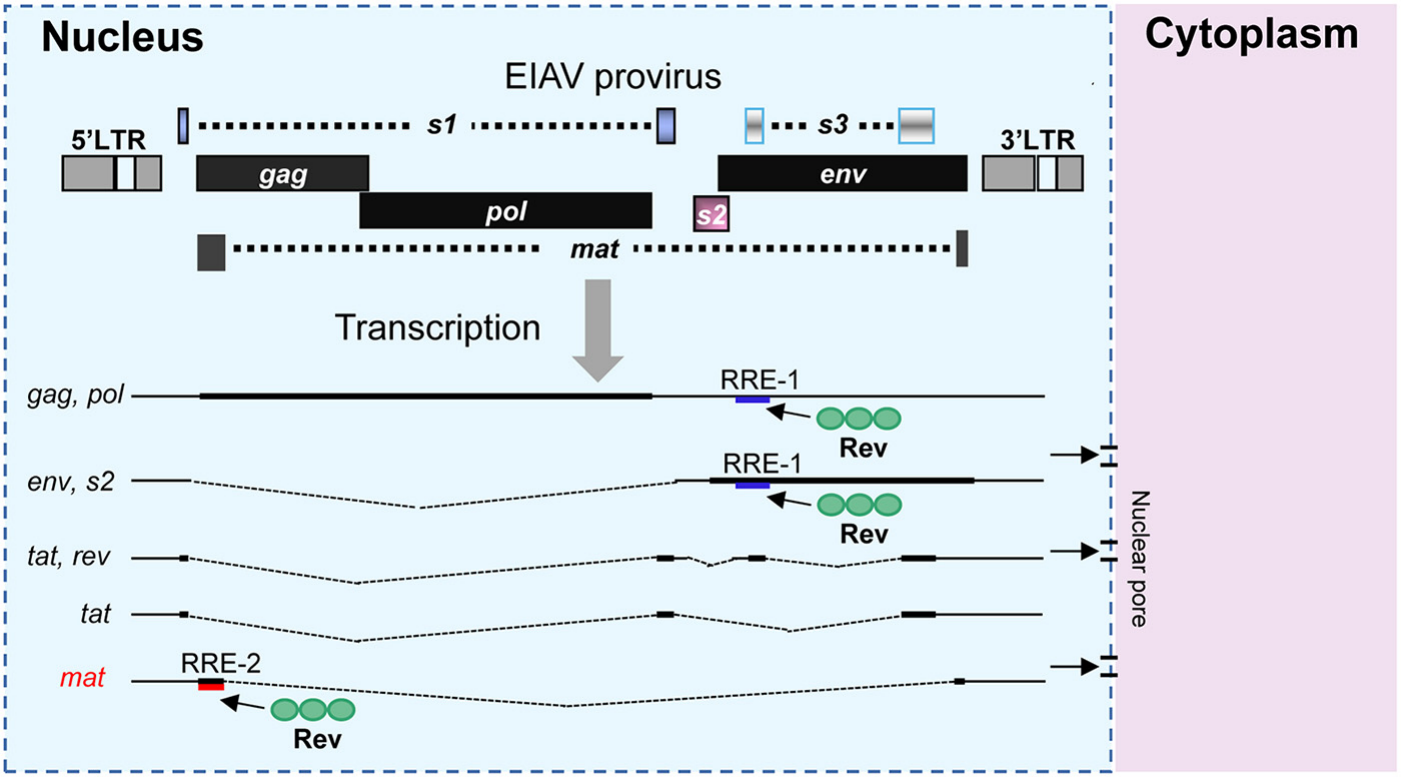
The EIAV proviral genome structure and transcriptional patterns. The previously identified Rev-response element (RRE) was named RRE-1 (blue line) and was in the env-coding region. The unspliced full-length mRNA transcripts encoding Gag and Pol proteins and an incompletely spliced transcript encoding Env and S2 proteins were exported to the cytoplasm in a pathway mediated by the binding of Rev to RRE-1. The transcripts encoding Tat or Rev protein were exported to the cytoplasm via an endogenous cellular pathway used by cellular mRNAs independent of Rev. The transcripts encoding Mat protein were exported to the cytoplasm via a pathway mediated by the binding of Rev to the first exon of Mat mRNA. The first exon of the mat, therefore, had the function of RRE and was named RRE-2 (red line).

Lentivirus transcription takes place in the nucleus, and the various transcripts produced by transcription usually undergo nuclear export via two pathways for gene expression (8). In the case of HIV-1, nuclear export of the completely spliced transcripts is mediated by cellular mRNA transporter proteins, while nuclear export of the unspliced full-length and incompletely spliced transcripts is mediated by Rev through binding to RRE via the CRM1 pathway. In EIAV, only one RRE has been found to date, and this is in the Env-coding region (29, 40). In this report, we found a novel fully spliced transcript in EIAV, encoding a 20 kDa protein, and named it Mat. Interestingly, we demonstrated that the expression of Mat depended on Rev, that Rev could increase the nuclear export of Mat mRNAs, and that Rev could specifically bind to Mat mRNAs. Furthermore, the first exon of Mat mRNAs (Mat-exon1 mRNA) has been identified as a key region for Rev-mediated nuclear export. These results indicated that, in addition to the reported previously RRE located in the Env-coding region in EIAV, there was a second RRE in the Mat coding region. These findings updated our knowledge of EIAV genome structure and its gene transcription pattern, as illustrated in Fig. 6.

In lentiviruses, alternative splicing is a common phenomenon that promotes the production of various transcripts encoding different viral proteins. Previous studies have shown that five major transcripts are produced from the EIAV genome (21, 22), encoding three structural proteins, three accessory proteins, and a protein with unknown function, Ttm. Except for the unspliced full-length transcripts, all spliced transcripts share a 5′-splice donor site. In this study, we report a novel EIAV transcript, which is produced by a single splicing event, with a 5′-splice donor site that is different from other reported splicing transcripts. This suggested that EIAV alternative splicing patterns were complex. Importantly, we confirmed that this transcript encodes a novel viral protein, here designated Mat. Although the biological function of Mat is unclear at present, our research results have further updated our knowledge of the genome structure of EIAV and its patterns of gene transcription.

Cis-acting repressing sequence (CRS) is a type of RNA sequence element that regulates gene expression by inhibiting the nuclear export of mRNAs (41). It is widely present in a variety of viruses, including HIV-1 (42–44), EIAV (45), HBV (46), and HPV (47), as well as in cellular transcripts. Although no uniformly recognizable sequence commonality was detected in CRS, it has been determined that a variety of host proteins could specifically bind to CRS and restrict RNA in the nucleus (48–50). In HIV-1, several CRS have been identified in multiple regions such as gag, pol, and env genes and it has been demonstrated that these inhibitory sequences could prevent nuclear export of unspliced and partially spliced transcripts, but the supply of Rev overcomes this inhibition (42, 44, 51). A previous study by Rosin-Arbesfeld et al. (45) also found that a CRS exists in the center of the EIAV genome (the C terminus of the Pol coding region) and demonstrated that this CRS could inhibit the nuclear export of viral transcripts. In this study, we found that the mat or mat-exon1gene constructs showed poor expression and cytoplasmic RNA accumulation in the absence of Rev, suggesting the existence of a potential CRS in the first exon of the mat gene. Interestingly, a CRS has been identified at the N terminus of the HIV-1 gag gene in a previous study (43). The first exon of the mat gene is also located at the N terminus of the EIAV gag gene. However, the gag genes of HIV-1 and EIAV lack sequence homology. Importantly, the expression of Mat or Mat-exon1 was rescued by coexpression with Rev, indicating that Rev may overcome the inhibitory effect of the mat-exon1 gene, which also suggested that mat-exon1 sequences contained an element enabling Rev-mediated nuclear export, such as an RRE. These results suggested that both a CRS and RRE may exist simultaneously in the mat-exon1, or the two may overlap each other. Notably, the overlap of a CRS and RRE has been documented in HIV-1 (51).

Interestingly, we found that the expression of Mat is dependent on Rev. Rev is an accessory protein necessary for lentiviral replication, and its biological function is to mediate the transport of the unspliced and incompletely spliced viral mRNAs from the nucleus to the cytoplasm. Mechanistically, Rev binds to an RRE located within the Env-coding region, then polymerization occurs, and the polymerized protein facilitates the export of the Rev-RNA complex into the cytoplasm through a CRM1-dependent pathway. We found that Rev could specifically bind to mat mRNAs, and that mutation of the Rev multimerization site disrupted its ability to mediate Mat expression. In addition, the use of the CRM1 inhibitor LMB significantly decreased Mat expression levels. These results demonstrated that the expression of Mat depended on the nuclear export activity of Rev. Previous studies have identified the RRE of EIAV that is in a 555 nt region near the 5′ end of the env gene (29, 40). In this study, we found that Rev could bind to Mat-exon1 mRNAs and mediate their nuclear export, and this phenomenon has been verified in 5 other EIAV strains. This indicates that the first exon (393 nt) of mat mRNAs is the second known RRE of EIAV. We named it RRE-2, with the reported RRE located in the Env coding region defined as RRE-1. In HIV-1, an additional RRE was also found in the packaging signal located in the 5′-untranslated region (5’UTR) of the genome (52, 53). Here, this HIV-1 RRE is termed 5’RRE. Mutation of 5’RRE led to reduced nuclear export of viral genomic RNA (gRNA) and decreased gRNA packaging efficiency but did not affect the production of viral proteins and the export of viral particles. However, replication of the virus was severely disrupted (53). Rem is an accessory protein encoded by mouse mammary tumor virus (MMTV) and is a homolog of the Rev protein in lentivirus. MMTV, a member of the genus Betaretrovirus, harbors two Rem-responsive elements (RmREs): a 5’RmRE located at the 5’UTR of gRNA that is required for nuclear export of unspliced RNA, and a 3′ RmRE located at env-U3 junction region that is needed for expression of both unspliced and spliced transcripts (54). Therefore, EIAV is different from HIV-1 and MMTV in that its two RREs are in the coding region sequence. We noticed that RRE-2 plays the nuclear export function of Rev-RRE only in the context of the mat transcript, but not in the context of gag/pol mRNA. However, RRE-1 could mediate transport of both Env-encoding transcripts and Gag/Pol-encoding transcripts. Possible explanations for this difference include that the nuclear export ability of the Rev-RRE-2 complex is weaker than that of Rev-RRE-1, or that the structure of RRE-2 may be more susceptible to interference from adjacent sequences.

We also observed that the nuclear export functions of Rev-RRE-1 and Rev-RRE-2 are dependent on different amino acid sites and regions of Rev. This suggested that the molecular mechanisms by which Rev mediated nuclear export of diverse target RNAs may differ, although to date no similar phenomenon had been reported in lentiviruses. We speculate that this may be related to the three-dimensional structure of Rev or the involvement of host factors (27, 34, 55).

In summary, we identified a novel protein, Mat, from EIAV, and demonstrated that the expression of Mat depended on Rev and that the first exon of mat mRNA acts as a Rev binding element to bind Rev and mediate Mat expression via the CRM1 pathway. These findings updated the EIAV genome structure, highlighted the diversification of posttranscriptional regulation patterns in EIAV, and provided important insights into the Rev-dependent nuclear export of completely spliced transcripts in lentiviruses.

## MATERIALS AND METHODS

### Plasmids.

The Mat expression plasmid, pcDNA-Mat-HA, was constructed by inserting the mat gene sequence into a pcDNA3.1 (+) vector and fusing HA-tags to the C-terminal. To construct a codon-optimized Mat expression plasmid, pcDNA-optMat-HA, we cloned the sequence corresponding to mat-exon1 from the codon-optimized Gag plasmid and inserted it before the mat-exon2 sequence. The plasmid pcDNA-Mat-exon1_FDDV_-HA was constructed by cloning the first exon of the mat gene from pCMV3-8 (56) and inserting it into pcDNA3.1-HA. The other 5 Mat-exon1 expression plasmids were synthesized according to the following EIAV strains genome sequence: EIAV_UK_ (GenBank accession number AF016316.1; nt450-836), EIAV_Miyazaki-2011-A_ (GenBank accession number JX003263.1; nt450-836), EIAV_Cornwall_ (GenBank accession number MH580898.1; nt231-617), EIAV_DE_ (GenBank accession number KM247554.1; nt114-500) and EIAV_NC-Dagmar_ (GenBank accession number MH820164.1; nt100-486). The Rev expression plasmid, VR-Rev-Flag, was constructed by inserting the EIAV Rev gene sequence into a VR1012 vector and fusing Flag-tags to the C-terminal. VR-Rev_HIV_-Flag was constructed by substituting the HIV-1 Rev gene sequence (GenBank accession number AF324493.2) into VR-Rev-Flag. A series of Rev-mutant plasmids were generated using site-directed mutagenesis on the template plasmid VR-Rev-Flag. The complete EIAV gag/pol gene was PCR amplified from the molecular clone pCMV3-8 and inserted into the pEGFP-N1 vector (Clontech, USA) to construct the plasmid pGP. The Rev-responsive element (RRE) was cloned from pCMV3-8 and inserted into the EcoRI and *Not*I sites of pGP to construct the pGP-RRE plasmid. The mat-exon1 sequence was cloned as an EcoRI-*Not*I fragment into pGP, which obtained plasmids containing a single mat-exon1(pGP-Mat/E1) as well as multimer copies of the mat-exon1(pGP-3×Mat/E1). All constructed plasmids were verified by sequencing.

### Cell culture and transfection.

Human embryonic kidney epithelium 293T (HEK293T) cells and HeLa cells were cultured in DMEM-high glucose (Sigma-Aldrich, USA) supplemented with 10% fetal bovine serum (Sigma-Aldrich, USA) and 1% penicillin-streptomycin (Gibco, USA). Equine monocyte-derived macrophages (eMDM) cells were isolated from fresh heparinized horse whole blood as previously described (57), and cultured in RPMI 1640 (Sigma-Aldrich, USA) supplemented with 20% donor equine serum (HyClone, USA) and 40% newborn bovine serum (Ausbian, Australia). Fetal donkey dermal (FDD) cell cultures were maintained in minimal essential medium (α-MEM, Gibco, USA) supplemented with 10% fetal bovine serum (Sigma-Aldrich, USA) and 1% penicillin-streptomycin (Gibco, USA) (58). All cells were maintained in a humidified incubator at 37°C containing 5% CO_2_.

Cells were transfected with the indicated plasmids using PolyJet DNA transfection reagent (SignaGen, USA), following the manufacturer’s instructions. EGFP-expression construct (pEGFP-N1) was added to all transfections to monitor transfection efficiency. All transfections were repeated at least three times.

### Amplification and identification of mat.

For the amplification of mat, polyadenylated RNAs were prepared from frozen horse tissues infected with EIAV_LN40_ using a TRIzol method (59). The tissues were homogenized in liquid nitrogen, and 1 mL of TRIzol reagent was used to incubate each sample. RNAs were precipitated with 0.5 mL isopropyl alcohol and then washed with 1 mL 75% ethanol to obtain purified total RNAs. For further identification of the mat transcript, an EIAV virulent strain (EIAV_DLV34_) or vaccine strain (EIAV_DLV121_) at equivalent titer was used to infect eMDM cells cultured in T25 flasks (57). At 4 days postinfection, cells were harvested and total RNAs were extracted using a Bio-fast simply P RNA extraction kit (Bioer, China).

About 1 μg of RNAs were reverse transcribed into cDNAs using a PrimeScript RT reagent kit with gDNA Eraser (TaKaRa, Japan) following the manufacturer’s protocol. EIAV transcripts were amplified with Kod FX Neo polymerase (Toyobo, Japan) in polymerase chain reactions (PCR). Nested primer pairs included: p399 (5′-GGACAGCAGAGGAGAACTTACAG-3′; forward primer), p8144 (5′-AAGGGACTCAGACCGCAGAATCT-3′; reverse primer)/p8081 (5′-TAAAAACAGGAASTTAACGCGTCAC-3′; reverse primer) were used to amplify EIAV transcripts. Nested primer pairs: p555 (5′-TCAAAAGCTAACTAATGGTAAC-3′; forward primer)/p7878 (5′-ATTGAGGCATTGTTACATGAGA-3′; forward primer), p643 (5′-CAATTAAGGGACGTCATTCCAT-3′; reverse primer)/p7855 (5′-GTAGCTGGATTTAGCATTTTCC-3′; reverse primer) were used to identify mat-specific transcripts. The amplification conditions were as follows: 2 min at 98°C; 35 cycles of the 30s at 98°C, 30s at 56°C and10 to 60s at 72°C; and a final extension step of 10 min at 72°C. PCR products were separated using electrophoresis on 1% agarose gels in 1×Tris-acetate-EDTA and were visualized by staining with ethidium bromide. The amplified fragments were purified using a gel extraction kit (Vazyme, China) and cloned into a pMD-18T vector (TaKaRa, Japan).

### Western blotting.

The expression of the indicated plasmids was analyzed using Western blotting as previously described (60). In brief, cells were harvested 48 hpt and resuspended in lysis buffer (150 mM Tris-HCl [PH 7.6], 50 mM NaCl, 5 mM EDTA, and 1% Triton X-100). Total protein extracts were separated by SDS-PAGE (Genscript, USA) and then blotted to NC membranes (Millipore, Germany). The membrane was subsequently blocked for 2 h with 5% skim milk (Biosharp, China) in PBS at room temperature. PBS containing 5% skim milk was used to dilute primary and secondary antibodies. After washing, the membrane was analyzed on the LI-COR Odyssey Imaging System (LI-COR, USA). All experiments were performed at least in triplicate.

### Antibodies.

Mat proteins in eMDM cells infected with EIAV_DLV121_ and EIAV_DLV34_ were detected using a mouse anti-Mat monoclonal antibody (1G3), which was produced by immunization of mice with a Keyhole Limpet Hemocyanin (KLH) carrier protein containing a polypeptide corresponding to the C-terminal 15 amino-acids (ENAKSSYISCNNASI) of Mat. Gag proteins were analyzed using a mouse anti-p26 MAb (9H8), kept in our lab (61). The primary antibodies purchased from commercial sources are listed below: mouse anti-HA MAb (Sigma, USA), mouse anti-FLAG MAb (Sigma, USA), mouse anti-GFP mouse MAb (Affinity Bioscience, USA), mouse anti-LaminB1 MAb (Proteintech, China), mouse anti-α tubulin (Abcam, UK), and mouse anti-β actin MAb (Sigma, USA). Goat anti-mouse IRD800-conjugated MAb (Sigma, USA) was used as a secondary antibody for Western blotting.

### Cell fractionation and quantitative PCR analysis.

For the preparation of nuclear and cytoplasmic RNAs, HEK293T cells were harvested 36 hpt and purified using a PARIS protein and RNA isolation kit (Invitrogen, Thermo Fisher Scientific, USA) according to the manufacturer’s protocols. Equal volume RNAs were treated with gDNA Eraser that has potent DNA degradation activity to remove genomic DNA contamination and then subjected to reverse transcription using the PrimeScript RT reagent kit with gDNA Eraser (TaKaRa, Japan) according to the manufacturer’s protocol. The distribution of mat mRNAs, GFP mRNAs, mat-exon1 mRNAs, or gag mRNAs in transfected cells was assessed using SBYR-Green (TaKaRa, Japan)-based quantitative PCR analysis on Aligent Mx3005P to calculate the absolute quantification with standard curves. The primers used were as follows: Mat-forward (5′-AAGGGACGTCATTCCATTGTT-3′) and Mat-reverse (5′-ATTTGTAAGCCCATCTTAACG-3′); GFP-forward (5′-GCAAGCTGACCCTGAAGTTCATC-3′) and GFP-reverse (5′-GTCTTGTAGTTGCCGTCGTCCTT-3′) Mat-exon1-forward (5′-AGTTAGAGAAGGTGACGGTAC-3′) and Mat-exon1-reverse (5′-CAACAATGGAATGACGTCCCT-3′); Gag-forward (5′-CGATGCCAAATCCTC CATTAG-3′) and Gag-reverse (5′-CTGATCAAAAGCAGGTTCCATCT-3′). One or two representative samples without reverse transcription were used as controls in each PCR analysis to ensure the elimination of transfected plasmid DNA. The distribution ratio of nuclear and cytoplasmic mat mRNA (GFP, mat-exon1, or gag mRNA) from the same sample was calculated essentially as described previously (35, 62).

### *In vitro* transcription and RNA-pulldown assay.

Template plasmids were digested using NotI (Thermo Scientific, USA) to generate linearized DNAs, which were subsequently transcribed to produce copies of the mRNAs using an *in vitro* transcription kit (New England Biolabs, UK) according to the manufacturer’s protocol. The equimolar mRNAs were labeled using biotin with an RNA 3′ End Desthiobiotinylation kit (Thermo Scientific, USA). The labeled mRNAs were then captured using streptavidin magnetic beads (Thermo Scientific, USA). After washing three times with Protein-RNA Binding Buffer (20 mM Tris [pH 7.5], 50 mM NaCl, 2 mM MgCl_2_, 0.1% Tween 20 Detergent), 100 μg of the total protein extracted from transfected cells were added to the RNA-bound beads and incubated for 1h on a roller at 4°C. Beads were then washed five times with Washing Buffer (20 mM Tris [pH 7.5], 10 mM NaCl, 0.1% Tween 20 Detergent), and the beads containing RNA-binding protein complexes were harvested and analyzed with Western blotting.

### Data availability.

The *mat* sequence reported in this study was deposited to GenBank genome database under accession number OM417133.
